# Effects of Eugenol on *Haemoproteus columbae* in domestic pigeons (*Columba livia domestica*) from Riyadh, Saudi Arabia

**DOI:** 10.1042/BSR20190409

**Published:** 2019-05-24

**Authors:** Dina M. Metwally, Razan A. Al-Talhi, Ibrahim A.H. Barakat, Manal F. ElKhadragy

**Affiliations:** 1 Zoology Department, College of Science, King Saud University, Riyadh, Kingdom of Saudi Arabia; 2 Parasitology Department, College of Veterinary Medicine, Zagazig University, Zagazig, Egypt; 3 Cell Biology Department, National Research Center, Dokki, Giza, Egypt; 4 Department of Zoology and Entomology, Faculty of Science, Helwan University, Cairo, Egypt

**Keywords:** Butalex®, Eugenol, Haemoproteus columbae

## Abstract

Eugenol was investigated for the treatment of *Haemoproteus columbae* (*H. columbae*) infected squabs (young domestic pigeons, *Columba domestica*). Thirty naturally-infected squabs were divided into three groups of 10 each. One group was treated with Eugenol, while the positive and negative control groups were administered buparvaquone (Butalex®) and distilled water, respectively. The number of infected red blood cells (RBCs) was calculated in all groups before and after treatment at 4-day intervals for 16 days. The results showed a significant therapeutic effect of Eugenol, with a progressive decrease in the number of infected RBCs from 89.20 ± 2.11 before treatment to 0.90 ± 0.31 at the end of treatment (*P*≤0.05). Butalex® was able to suppress the number of infected RBCs from 93.70 ± 1.72 before treatment to 0.90 ± 0.35 at the end of the experiment (*P*≤0.05). Eugenol showed therapeutic effects against *H. columbae* and may be regarded as a candidate for further studies to develop new drugs against blood parasites, in both animals and humans.

## Introduction

Pigeons of the order Columbiformes, mostly domestic pigeons (*Columba livia domestica*), are found in most towns and cities worldwide [1]. These pigeons have a role in spreading zoonoses to humans and are also reservoirs for many parasitic infections that they transmit to other birds [2,3]. The genus *Haemoproteus* is distributed worldwide and is a vector-borne blood parasite. *Haemoproteus columbae* is the causative agent of pigeon malaria and is transmitted to pigeons via the pigeon louse fly, *Pseudolynchia canariensis*, which transmits the disease by inoculating the infective sporozoites. Schizogony occurs in the lung and liver endothelia, resulting in the release of merozoites, which then invade erythrocytes and develop into gametocytes. Gametocytes are visible in blood smears and partially surround the nucleus of RBCs [4]. Under some research conditions, the *Haemoproteus* spp. infection has been considered mild or even nonpathogenic in birds. Nowadays, it is well understood that Haemoproteus can affect avian body mass, immune and reproductive systems, and community relationships and may lead to the death or extinction of more susceptible bird species [5–10]. The resistance of some blood parasites to standard drugs has prompted scientists to search for more efficient drugs with new mechanisms of action [11]. Herbal extracts (e.g., quinine and artemisinin) have been a valuable source of new drugs, especially anti-hemosporidial agents [12]. Some plant products have been shown to be useful as anti-hemosporidial agents [11,13,14]. To our knowledge, no documented research has studied the effects of Eugenol against *H. columbae*. Eugenol is the main component of cloves and has a wide range of applications in perfumes, flavorings, essential oils, and in medicine as a local antiseptic and anesthetic [15]. Some previous studies have shown that Eugenol has inhibitory activities against different parasitic agents, such as gastrointestinal helminths [16,17] and leishmania [18]. Thus, the objective of the present study was to evaluate the effects of Eugenol against *H. columbae* in the domestic pigeon and compare these effects with those of the traditional drug, buparvaquone (Butalex®).

## Materials and methods

### Ethics statement

The present study was approved by the Institutional Committee of Post-Graduate Studies and Research at King Saud University (Saudi Arabia). All efforts were made to minimize suffering.

### Animals

The present study used squabs of domesticated pigeons (*C. livia domestica*) from the Shaqraa region of Riyadh, Saudi Arabia. Thirty squabs, 3–4 weeks old and weighing 450–500 g, were used. All squabs were kept separately in metal cages, with free access to food and water.

Blood smears were obtained from all squabs to examine blood-borne parasites. Oral examination was performed to detect *Trichomonas* spp. and fecal examination to detect internal parasites (helminths and protozoa). Feathers were examined by naked eye and lens to search for any external parasites. Blood smears revealed the presence of *H. columbae. Pseudolynchia canariensis* (*P. canariensis*) was detected adherent to the squabs’ skin, under the wings. Squabs were not treated for any of these infections prior to the study. All squabs included in the study were free of helminths and protozoal infections.

### Source of Eugenol

Eugenol (2-methoxy-4-(2-propenyl) phenol, 4-allyl-2-methoxyphenol, 4-allylguaiacol) was obtained in liquid (99%) form from Sigma-Aldrich.

### Experimental design

**Figure 1:**
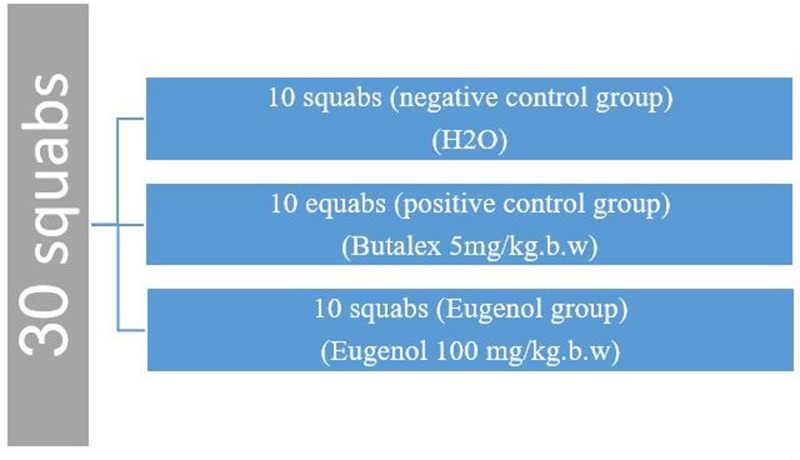
Experimental groups

Infected squabs were randomly divided into three groups of 10 each (Figure 1). The first group was treated with distilled water and considered as the negative control group. The other two infected groups were treated with Butalex® (positive control group) or Eugenol (Eugenol group). Butalex® was administered intramuscularly as a single recommended dose (5 mg/kg body weight) [19]. The Eugenol (100 mg/kg body weight) was administered by oral route once a day for 16 days [20]. The parasitemia rate (infected RBCs counts) was calculated for all groups on days 0, 4, 8, 12, and 16. Biochemical analysis of blood samples [total serum protein, bilirubin, albumin, glucose, aspartate aminotransferase (AST), and alanine aminotransferase (ALT)] and histopathological examination of lung and liver tissues were performed at the end of the experimental period (16 days).

### Parasitemia rate and biochemical analysis

Blood samples were collected either in tubes containing ethylenediaminetetraacetic acid (EDTA) as an anticoagulant agent for the preparation of blood smears or in normal tubes without EDTA for serum separation and subsequent biochemical analyses. Blood samples were obtained from the brachial vein punctured by a lancet and smears were prepared on clean microscopic slides, fixed with absolute methanol, and then stained with 10% aqueous Giemsa stain for 45 min. Five hundred RBCs were counted for each animal at 4-day intervals for 16 d, to calculate the parasitemia rate after each treatment [21]. Serum was separated by centrifugation at 1800 × ***g*** for 10 min and stored at −20°C until analysis. Bilirubin, albumin, glucose, AST, and ALT concentrations were measured by commercial kits (United Diagnostic Industry, Dammam, Saudi Arabia) using a BAS 3000 semi-automatic biochemistry analyzer (Labomed, Los Angeles, CA, U.S.A.) and an Ultrospec 2100® UV-visible spectrophotometer (Biochrom, Holliston, MA, U.S.A.).

### Histopathological examination

On day 17, 24 h after the final treatment, the squabs were slaughtered by the Islamic method (Controlled slaughter) [22]. Transverse sections were prepared from squab liver and lung tissues according to the method described by Luna [23]. Briefly, liver and lung specimens were fixed with 10% formol saline, dehydrated with ethyl alcohol, cleaned with xylene, and embedded in paraffin wax. Five micron-thick paraffin sections were obtained using a rotatory microtome and were stained with hematoxylin and eosin (H&E).

### Statistical analysis

Data were analyzed using the SPSS statistical program (version 22; IBM, Armonk, NY, U.S.A.). RBCs infection data were analyzed by two-way ANOVA and blood biochemistry data were analyzed by one-way ANOVA. Comparisons between treatment means were performed using Duncan’s test and results were considered significant at *P*≤0.05. Results were expressed as mean ± standard error of mean (SEM).

## Results

### Examination of blood smears

#### Negative control group

**Figure 2:**
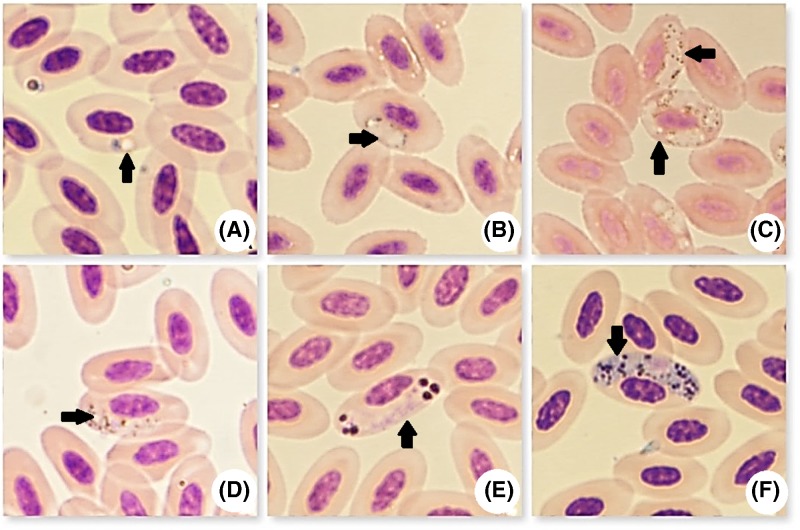
Microphotographs of *H. columbae* stages (black arrows, Giemsa stain, ×1000) (**A**) Trophozoites in ring form, (**B**) growing gametocytes, (**C**) multiple gametocyte infection, (**D**) immature gametocytes, (**E**) fully formed micro-gametocytes, and (**F**) fully formed macro-gametocytes.

*H. columbae* was detected in the RBCs of infected pigeons (Figure 2). Growing trophozoites formed signet ring-like parasites, characterized by a thin layer of cytoplasm surrounding a vacuole, with a compact nucleus to one side (Figure 2A). Developmental stages, in the form of small dots, were seen initiating growth and they were situated lateral to the RBCs nucleus. Some also initiated growth in polar or sub-polar positions (Figure 2B). Multiple infections were seen, with up to five trophozoites per RBCs (Figure 2C). Immature gametocytes were elongated laterally to the RBCs nucleus and sometimes assumed a cap-like position. The entire margins of the parasites were rounded or sometimes with pointed ends (Figure 2D).

Gamonts were seen partially encircling the nucleus of the host cell, forming a halter and measuring 10.625–12.575 µm in length, with a mean of 11.6 µm and 2.226–3.8 µm in width, with a mean of 3.013 µm. Micro-gamonts were stained blue to pink. Their nuclei were stained pink and were diffused, while pigment granules were collected into spherical masses at both poles (Figure 2E). Macro-gamonts were stained dark blue, with a compact nucleus stained dark purple to red. Macro-gamont pigment granules were dispersed throughout the cytoplasm (Figure 2F).

#### Positive control (Butalex®) and Eugenol groups

**Figure 3:**
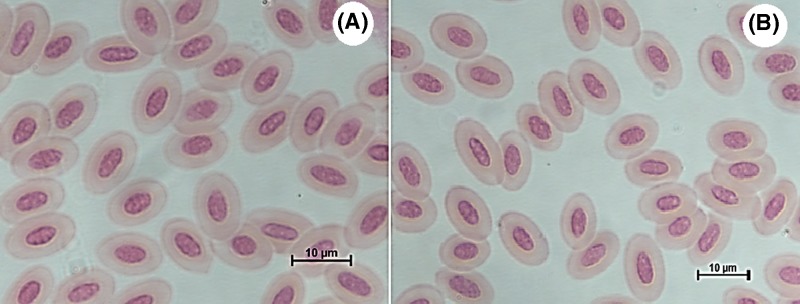
Blood smears from treated pigeons (**A**) The positive control (Butalex®) and (**B**) Eugenol-treated groups showed RBCs free of halter-shaped mature gametocytes in the cytoplasm (Giemsa stain, ×1000).

Examination of blood smears on day 17, from positive control (Butalex®, Figure 3A) and Eugenol-treated groups (Figure 3B) showed that the cytoplasm of RBCs was free of gametocytes.

### Parasitemia rate

The therapeutic effects of both Butalex® and Eugenol on *H. columbae* were evaluated in RBCs (Table 1). Results showed a significantly higher number of infected RBCs in the negative control group than in the other two groups. There was no significant difference in the number of infected RBCs between the Eugenol and positive control groups. Butalex® was the best treatment, since it had the lowest mean number of infected RBCs (39.52 ± 4.84).

**Table 1:** Effect of different treatments on the number of infected RBCs in squabs

Parameters treatment	Infected RBCs (%)	Noninfected RBCs (%)
Negative control (untreated)	99.66 ± 1.24 (20.03)^a^	399.84 ± 1.26 (79.97)^b^
Positive control (Butalex®)	39.52 ± 4.84 (7.90)^b^	458.48 ± 5.73 (7.90)^a^
Eugenol	42.80 ± 5.11 (8.56)^b^	457.02 ± 5.08 (91.44)^a^
Sig.	0.0000	0.0000

Mean values in the same columns with different superscripts (a, b) differ significantly from each other (*P*≤0.05).

Results of the analysis of the interaction effect between treatment and time by two-way ANOVA are shown in Table 2 and Supplementary Figure 1. The number of infected RBCs decreased with increasing treatment time, with the lowest infection rate observed on day 16 in all three experimental groups. Moreover, the peak of infection occurred on day 4 in the negative control group (110.90 ± 2.23). There were no significant differences in the number of infected RBCs between Butalex®- and Eugenol-treated squabs at days 0, 8, 12, and 16. However, at day 4, there were significantly fewer infected RBCs in the Eugenol-treated group but differ from each other on the fourth day. From the above results, we concluded that treatment with Eugenol provided equal effect on the parasite to Butalex®.

**Table 2:** Effect of the interaction between time and treatment on the number of infected RBCs in squabs

Treatment		Infected RBCs	Noninfected RBCs
Negative control (untreated)	Day 0	100.70 ± 2.44^b^	399.30 ± 2.44^efg^
	Day 4	110.90 ± 2.23^a^	388.40 ± 2.24^g^
	Day 8	102.00 ± 1.13^b^	397.20 ± 1.25^fg^
	Day 12	94.40 ± 0.96^c^	404.60 ±0.97^ef^
	Day 16	90.30 ± 0.99^c^	409.70 ±0.99^e^
Positive control (Butalex®)	Day 0	93.70 ± 1.72^c^	406.30 ± 1.72^ef^
	Day 4	74.30 ± 2.27^d^	425.70 ± 2.27^d^
	Day 8	30.40 ± 1.89^f^	469.60 ± 1.89^c^
	Day 12	14.70 ± 0.97^g^	485.30 ± 0.97^b^
	Day 16	0.90 ± 0.35^h^	498.20 ± 0.98^a^
Eugenol	Day 0	89.20 ± 2.11^c^	400.80 ± 11.81^ef^
	Day 4	68.10 ± 1.38^e^	431.90 ±1.38^d^
	Day 8	26.30 ± 1.76^f^	473.70 ± 1.76^c^
	Day 12	13.10 ± 1.30^g^	486.90 ± 1.30^b^
	Day 16	0.90 ± 0.31^h^	499.10 ± 0.31^a^
Sig.		0.0000	0.0000

Mean values in the same columns with different superscripts (a, b, c, d, e, f, g, h) differ significantly from each other at *P*≤0.05.

### Biochemical analysis

Alterations in serum biochemistry parameters after treatment are shown in Table 3. There was no significant difference in serum ALT concentration between the positive control and Eugenol group; however, serum ALT concentration in both of these groups was significantly lower than in the negative control group, which gave the highest concentration (56.95 ± 1.11 IU/L). There was also a significant difference in AST enzyme levels between groups, with the highest mean level of 384.35 ± 0.25 IU/L in the negative control group and the lowest mean level of 178.03 ± 0.45 IU/L in the Eugenol group. Albumin and glucose levels were significantly different between all groups. The highest mean albumin concentration (1.20 ± 0.05 g/dl) was observed in the Eugenol group, followed by the positive control group (1.00 ± 0.03 g/dl) and the negative control group (0.85 ± 0.00 g/dl). However, the inverse trend was observed for serum glucose concentration. There were no significant differences in bilirubin levels between the negative and positive control groups. However, bilirubin levels were significantly lower in the Eugenol group (0.11 ± 0.02 g/dl) than both the negative and positive control groups. In general, there was a significant difference between groups treated with Butalex® and Eugenol for all serum parameters, except ALT enzyme levels. With the exception of total bilirubin concentration, all serum parameters in the positive control and Eugenol groups were significantly different from those in the negative control group.

**Table 3:** Effects of different treatments on serum biochemical parameters in squabs

Parameters group	ALT (IU/L) (g/dl)	AST (IU/L)	Albumin (g/dl)	Glucose (g/dl)	Total bilirubin (g/dl)
Negative control	56.95 ± 1.11^a^	384.35 ± 0.25^a^	0.85 ± 0.00^c^	348.04 ±0.30^a^	0.44 ± 0.10^a^
Positive control (Butalex®)	48.30 ± 3.46^b^	270.95 ±0.23^b^	1.00 ± 0.03^b^	336.35 ± 0.29^b^	0.42 ± 0.1^a^
Eugenol	44.20 ± 3.62^b^	178.03 ±0.45^c^	1.20 ± 0.05^a^	313.74 ±0.20^c^	0.11 ± 0.02^b^

Mean values in the same columns with different superscripts (a, b, c) differ significantly from each other at *P*≤0.05.

### Histopathological examination

#### Examination of lung tissue

**Figure 4:**
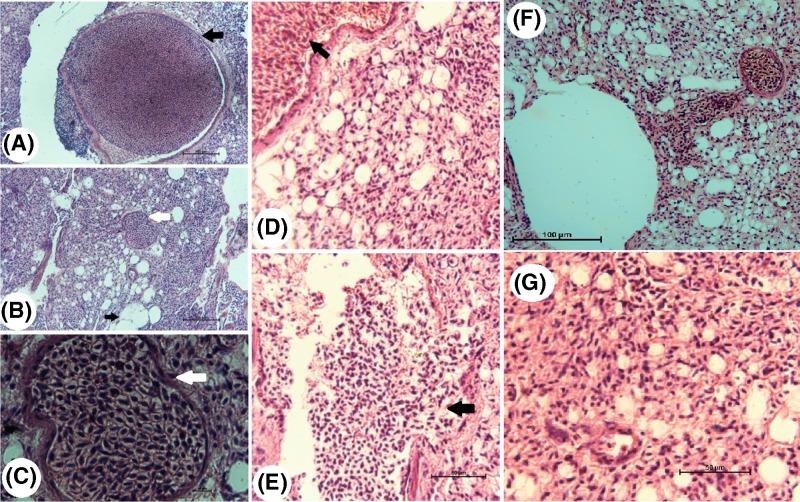
Photomicrographs of lung tissue sections from infected (**A–E**) and treated (**F** and **G**) squabs (**A**) Large schizont invading the endothelium lining of the lung tissue from squabs infected with *H. columbae* (black arrow, H&E, ×20), (**B**) small schizont (white arrow, H&E, ×20), (**C**) high magnification of B (white arrow, H&E, ×100), (**D**) schizonts in the endothelial cells of lung blood vessels (black arrow, H&E, ×40), (**E**) schizonts are ruptured and merozoites are disseminated in between the lung cells (black arrow, H&E, ×40), (**F**) schizonts were observed invading the blood vessel endothelium in squabs treated with Butalex® (H&E, ×20) and (**G**) schizonts were not seen in lung tissue sections of squabs treated with Eugenol (H&E, ×20).

Examination of lung tissue sections from infected squabs revealed the presence of large schizonts of the parasite in the wall of the lung blood vessels (Figure 4A) (black arrow) and also small schizonts (Figure 4B) (white arrow). The parasite does not invade the lung cells thus; the pathogenesis is mild (no eosinophils). Magnification of the schizonts showed that they contained merozoites (Figure 4C,D). After that, schizonts are ruptured and large merozoites are disseminated between the cells (Figure 4E). Examination of lung tissue sections from infected squabs treated with Butalex® revealed the presence of schizonts of the parasite (Figure 4F), but we were unable to determine if the schizonts were active. Schizonts were not affected by treatment with Butalex®. Schizonts of the parasite were not observed upon examination of lung tissue sections from infected pigeons treated with Eugenol (Figure 4G).

#### Examination of liver tissue

**Figure 5:**
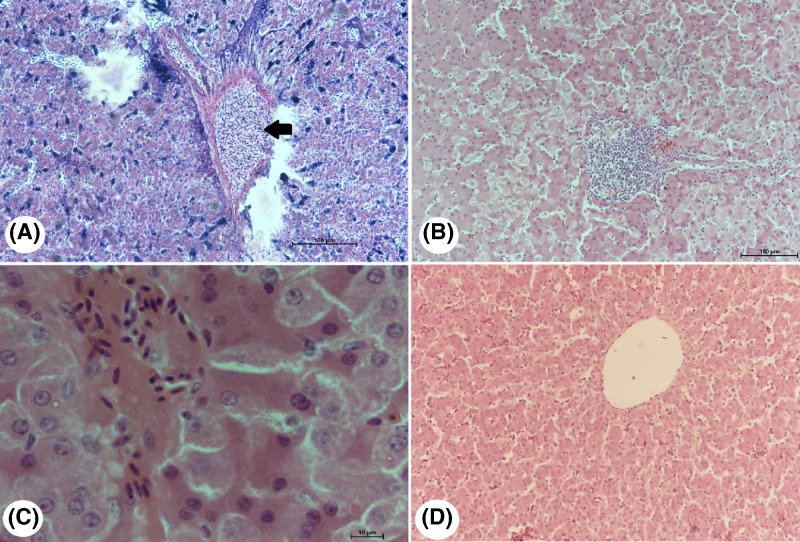
Photomicrographs of liver sections from infected and treated squabs (**A**) Schizonts (black arrow) were observed in hepatic endothelial cells, (**B**) schizonts (black arrow) (H&E, ×20) and (**C**) merozoites (H&E, ×100) were present within the hepatic tissue of squabs treated with Butalex® (white arrow). (**D**) Schizonts were not seen in liver tissue sections of squabs treated with Eugenol (H&E, ×20).

Examination of liver tissue sections from infected squabs showed that the schizonts of *H. columbae* inhabit the blood vessels of the liver and not invading the hepatic cells thus the pathogenesis on the liver is mild (no eosinophils) (Figure 5A). Examination of liver tissue sections of the Butalex®-treated squabs showed the presence of schizonts of the parasite and merozoites within the liver tissue, but it was difficult to determine if the merozoites were active or inactive (Figure 5B,C). Schizonts of the parasite were not seen upon examination of liver tissue sections from squabs treated with Eugenol (Figure 5D).

## Discussion

The phylum Apicomplexa contains a significant and diverse group of protozoan parasites. The genus *Haemoproteus* includes species that parasitize many species of domestic and wild birds [24].

*H. columbae* is known as the causative agent of pigeon malaria and is transmitted to pigeons via the pigeon louse fly, *P. canariensis*, which transmits the disease by inoculating infective sporozoites. Schizogony occurs in the lung and liver endothelium, resulting in the release of merozoites. The merozoites then invade red blood cells (RBCs) and develop into gametocytes. Gametocytes are visible in blood smears and partially surround the nuclei of RBCs [4]. *Haemoproteus* spp. infections are mild and they are nonpathogenic parasites in some birds [5,6]. However, *Haemoproteus* spp. can affect avian body mass [7], immune and reproductive systems [8], and community relationships [9] and may lead to the death or extinction of more susceptible bird species [10].

Many synthetic drugs, such as Butalex®, are used to treat hemoparasites, but they are expensive, have narrow margins of safety, and are susceptible to drug resistance [25]. To reduce the side effects of common drugs, some studies have investigated the use of natural drugs [26,27]*.*

Plant-derived products, such as essential oils and methanolic extracts, are potential alternative agents to synthetic chemical products [28]. These substances are commonly used to maintain the social life of animals and to overcome issues of food safety and economics, because they are generally cost-effective and safe for both humans and animals [29]. Some efforts have been made to find more effective anti-hemosporidial compounds and some herbal products have been reported to be effective anti-malarial therapies [13]. Some previous studies have shown that Eugenol has inhibitory effects against different parasitic agents, such as Giardia [30], leishmanial, and gastrointestinal helminths [31–33].

In the present study, the therapeutic activity of Eugenol (100 mg/kg body weight) against *H. columbae* in naturally-infected squabs, was investigated*.* The results showed that Eugenol has a significant inhibitory effect on *H. columbae*, in both gametocytes and schizonts. Treatment with Eugenol was not significantly different from treatment with Butalex®, in terms of decreasing parasitemia at the end of the treatment period. Our data showed a time-dependent pattern for the therapeutic effect of Eugenol. At the beginning of the study, the numbers of infected RBCs in the Butalex® and Eugenol groups were 93.70 ± 1.72 and 89.20 ± 2.11, respectively, which decreased to 0.90 ± 0.35 and 0.90 ± 0.31, respectively, on post-treatment day 16 (*P* ≤ 0.05). Both Butalex® and Eugenol showed cidal activity against *H. columbae* gametocytes on day 16 after treatment, with all blood films being free of gametocytes.

Serum AST, ALT, albumin, total bilirubin, and glucose levels were all significantly different between negative control and treated groups. There were no significant differences in ALT levels between the Butalex®- and Eugenol-treated groups. However, both of these groups had significantly lower levels of serum ALT than the negative control group, which showed the highest levels at 56.95 ± 1.11 IU/L. There were significant differences in AST enzyme levels between groups, with the highest levels seen in the negative control group (384.35 ± 0.25 IU/L) and the lowest in the Eugenol group (178.03 ± 0.45 IU/L). High levels of AST and ALT in the negative control group may be due to liver cell damage caused by hepatic migration of parasites as part of their life cycle, which is characterized by injury to numerous hepatocytes. This result was in accordance with previous reports [34]. High levels of AST in the Butalex®-treated group may have been due to the presence of schizonts in the liver.

High levels of albumin may also occur due to the impairment of liver cells by hepatic migration of parasites. Most reports on the pathogenicity of avian hemoparasites have been based on evidence of high parasitemia in sick birds [35,36], but there have been no reported studies comparing biochemical parameters between parasitized and treated birds. However, Ots and Horak reported that serum albumin levels were not affected by *Haemoproteus* infection in great tits [37]. They concluded that *Haemoproteus* infection does not induce a severe inflammatory response and infected individuals do not reveal symptoms of poor nutritional state. Conversely, hypoalbuminemia in pigeons infected with *Haemoproteus* were reported [34]. However, Eugenol group reported low level of bilirubin. Hypoglycemia in the negative control group may be due to the erythrocytic phase of *H. columbae*, which consumes large amounts of glucose, up to 100 times more than consumed by uninfected RBCs [38]. *H. columbae*-induced infection, as indicated by erythrocytic activity, leads to severe anemia and reduced survival of birds, by increasing their predation risk under natural conditions. However, no reported studies have compared glucose levels between parasitized and treated birds.

In the present study, histopathological investigation revealed schizonts of the parasite in the studied organs of the negative group. They were seen invading both the lungs and the liver, in the endothelial lining of the blood vessels in infected squabs. The observed schizonts were thick-walled and variable in shape, resembling the schizont morphology of *Haemoproteus* species described by other researchers [39–41].

The variability in schizont shape in different tissues may have been related to the schizont attempting to conform to the local histological structures at the site of development. The free extravascular merozoites that were observed were most likely a result of damage to the vascular walls caused by the effect exerted by the large-sized parasitic stages [41].

The presence of schizonts in the endothelial lining of blood vessels in the lungs and liver of Butalex®-treated squabs indicates that Butalex® may have a static effect on schizonts, but a cidal effect on intra-erythrocytic gametocytes. To confirm this, blood samples would need to be examined 30 days after treatment to determine if intra-erythrocytic gametocytes reappear.

Schizonts were not detected in endothelial cells of liver and lung tissue blood vessels from Eugenol-treated squabs. This indicated that Eugenol had a significant cidal effect on schizonts. In the present study, Eugenol exhibited remarkable anti-*Haemoproteus* properties. This activity may be attributed to the presence of phenolic and flavonoid compounds, which are known to have cidal effects on both gametocyte and schizont stages, while Butalex® only has a cidal effect on gametocytes. Therefore, Eugenol may be the drug of choice for the treatment of *H. columbae* in pigeons. The present study demonstrates the need for additional studies to determine the exact mechanism (s), mode (s) of action, and probable side effects of Eugenol.

## Supporting information

**Supplementary Figure S1:** Mean values interaction between treatment and times on the infected red blood cells number in of squabs.

## Abbreviations

ALT: Alanine Aminotransferase
AST: Aspartate Aminotransferase
EDTA: Ethylenediaminetetraacetic acid
H&E: Hematoxylin and Eosin
RBCs: Red Blood Cells

## References

[1] Sandra M.T.M., Rosileia M.D.C., Cintia J.D.S. and Marisa B. (2007) Parasites of pigeons (*Columba livia*) in urban areas of Lages. Southern Brazil Parasitol. Latinoam. 62, 183–187

[2] Sari B., Karatepe B., Karatepe M. and Kara M. (2008) Parasites of domestic (*Columba livia domestica*) and wild (*Columba livia livia*) pigeons in Niğde. Turkey Bull. Vet. Inst. Pulawy 52, 551–554

[3] Radfar M.H., Khedri J., Adinehbeigi K., Nabavi R. and Rahmani K. (2012) Prevalence of parasites and associated risk factors in domestic pigeons (*Columba livia domestica*) and free-range backyard chickens of Sistan region, east of Iran. J. Parasit. Dis. 36, 220–225 10.1007/s12639-012-0112-5 24082532 PMC3427665

[4] Valkiunas G. (2004) Avian Malaria Parasites and Other Haemosporidia, pp. 12–16, CRC Press, Taylor & Francis, London

[5] Ashford R.W. (1971) Blood parasites and migratory fat at Lake Chad. IBIS 113, 100–101 10.1111/j.1474-919X.1971.tb05127.x

[6] Garvin M., Homer B. and Greiner E. (2003) Pathogenicity of *Haemoproteus danilewskyi*, Kruse, 1890, in blue jays (*Cyanocitta cristata*). J. Wildl. Dis. 39, 161–169 12685080 10.7589/0090-3558-39.1.161

[7] Valkiunas G., Zickus T., Shapoval A. and Iezhova T. (2006) Effect of *Haemoproteus belopolskyi* (Haemosporida: Haemoproteidae) on body mass of the blackcap *Sylvia atricapilla*. J. Parasitol. 92, 1123–1125 10.1645/GE-3564-RN.1 17152968

[8] Tomas G., Merino S., Moreno J., Morales J. and Martinez-De La Puente J. (2007) Impact of blood parasites on immunoglobulin level and parental effort: a medication field experiment on a wild passerine. Funct. Ecol. 21, 125–133 10.1111/j.1365-2435.2006.01214.x

[9] Ricklefs R., Fallon S. and Bermingham E. (2004) Evolutionary relationships, cospeciation, and host switching in avian malaria parasites. Syst. Biol. 53, 111–119 10.1080/10635150490264987 14965906

[10] Atkinson C., Dusek R., Woods K. and Iko W. (2000) Pathogenicity of avian malaria in experimentally-infected Hawaii Amakihi. J. Wildl. Dis. 36, 197–201 10.7589/0090-3558-36.2.197 10813599

[11] Muregi F.W., Chhabra S.C., Njagi E.N.M., Lang’at-Thoruwa C.C., Njue W.M., Orago A.S. (2003) *In vitro* antiplasmodial activity of some plants used in Kisii, Kenya against malaria and their chloroquine potentiation effects. J. Ethnopharmacol. 84, 235–239 10.1016/S0378-8741(02)00327-6 12648820

[12] Gessler M.C., Nkunya M.H., Mwasumbi L.B., Heinrich M. and Tanner M. (1994) Screening Tanzanian medicinal plants for antimalarial activity. Acta Trop. 56, 65–77 10.1016/0001-706X(94)90041-8 8203297

[13] Okeola V., Adaramoye O., Nneji C., Falade C., Farombi E. and Ademowo O. (2011) Antimalarial and antioxidant activities of methanolic extract of *Nigella sativa* seeds (black cumin) in mice infected with *Plasmodium yoelli nigeriensis*. Parasitol. Res. 108, 1507–1512 10.1007/s00436-010-2204-4 21153838

[14] Rodrigues J. and Gamboa N. (2009) Effect of dequalinium on the oxidative stress in *Plasmodium berghei*-infected erythrocytes. Parasitol. Res. 104, 1491–1496 10.1007/s00436-009-1355-7 19205739

[15] Jaganathan S.K. and Supriyanto E. (2012) Antiproliferative and molecular mechanism of Eugenol-induced apoptosis in cancer cells. Molecules 17, 6290–6304 10.3390/molecules17066290 22634840 PMC6268974

[16] Asha M.K., Prashanth D., Murali B., Padmaja R. and Amit A. (2001) Anthelmintic activity of essential oil of *Ocimum sanctum* and Eugenol. Fitoterapia 72, 669–670 10.1016/S0367-326X(01)00270-2 11543966

[17] Pessoa L.M., Morais S.M., Bevilaqua C.M.L. and Luciano J.H.S. (2002) Anthelmintic activity of essential oil of *Ocimum gratissimum* Linn. and Eugenolagainst *Haemonchus contortus*. Vet. Parasitol. 109, 59–63 10.1016/S0304-4017(02)00253-4 12383625

[18] Ueda-Nakamura T., Mendonça-Filho R.R., Morgado-Díaz J.A., Maza P.K., Dias Filho B.P., Cortez D.A.G. (2006) Antileishmanial activity of Eugenol-rich essential oil from *Ocimum gratissimum*. Parasitol. Int. 55, 99–105 10.1016/j.parint.2005.10.006 16343984

[19] El-Metenawy T.M. (1999) Therapeutic effects of some antihaematozoal drugs against *Haemoproteus columbae* in domestic pigeons. DTW Dtsch. Tierarztl. Wochenschr. 106, 72–72 10085582

[20] Paula-Freire L.I.G., Molska G.R., Andersen M.L. and de Araujo Carlini E.L. (2016) *Ocimum gratissimum* essential oil and its isolated compounds (Eugenol and myrcene) reduce neuropathic pain in mice. Planta Med. 82, 211–216 26584457 10.1055/s-0035-1558165

[21] Razavi S., Asadpour M., Malekpour S. and Jafari A. (2018) The field efficacy of *Nigella sativa* and *Berberis vulgaris* methanolic extracts against *Haemoproteus columbae*. Avicenna J. Phytomed. 8, 114 29632842 PMC5885325

[22] Qasmi Q.M. (2009) The Islamic Concept of Animal Slaughter, Dar al-Kotob al-Ilmiyah

[23] Luna L.G. (1968) Manual of histologic stainig methods of theArmed Forces Institute of Pathology (No. Sirsi) a385158). Armed Forces Institute of Pathology, USA

[24] Knowles S.C.L., Palinauskas V. and Sheldon B.C. (2010) Chronic malaria infections increase family inequalities and reduce parental fitness: experimental evidence from a wild bird population. J. Evol. Biol. 23, 557–569 10.1111/j.1420-9101.2009.01920.x 20070458

[25] Cheesman S.J. (2000) The topoisomerases of protozoan parasites. Parasitol. Today 16, 277–281 10.1016/S0169-4758(00)01697-5 10858645

[26] Rahman NNNA, Furuta T., Takane K. and Mohd M.A. (1999) Antimalarial activity of extracts of Malaysian medicinal plants. J. Ethnopharmacol. 64, 249–254 10.1016/S0378-8741(98)00135-4 10363840

[27] Ahmad A., Husain A., Mujeeb M., Khan S.A., Najmi A.K., Siddique N.A. (2013) A review on therapeutic potential of *Nigella sativa*: a miracle herb. Asian Pac. J. Trop. Biomed. 3, 337–352 10.1016/S2221-1691(13)60075-1 23646296 PMC3642442

[28] El Zalabani S.M., El-Askary H.I., Mousa O.M., Issa M.Y., Zaitoun A.A. and Abdel-Sattar E. (2012) Acaricidal activity of *Swietenia mahogani* and *Swietenia macrophylla* ethanolic extracts against Varroa destructor in honeybee colonies. Exp. Parasitol. 130, 166–170 10.1016/j.exppara.2011.10.013 22101075

[29] Razavi S.M., Asadpour M., Jafari A. and Malekpour S.H. (2015) The field efficacy of *Lepidium latifolium* and *Zataria multiflora* methanolic extracts against Varroa destructor. Parasitol. Res. 114, 4233–4238 10.1007/s00436-015-4661-2 26342827

[30] Machado M., Dinis A.M., Salgueiro L., Custodio J.B., Cavaleiro C. and Sousa M.C. (2011) Anti-Giardia activity of *Syzygium aromaticum* essential oil and eugenol: effects on growth, viability, adherence and ultrastructure. Exp. Parasitol. 127, 732–739 10.1016/j.exppara.2011.01.011 21272580

[31] Asha M.K., Prashanth D., Murali B., Padmaja R. and Amit A. (2011) Anthelmintic activity of essential oil of *Ocimum sanctum* and eugenol. Fitoterapia 72, 669–670 10.1016/S0367-326X(01)00270-2 11543966

[32] Pessoa L.M., Morais S.M., Bevilaqua C.M.L. and Luciano J.H.S. (2002) (2002). Anthelmintic activity of essential oil of *Ocimum gratissimum* Linn. and Eugenolagainst *Haemonchus contortus*. Vet. Parasitol. 109, 59–63 10.1016/S0304-4017(02)00253-4 12383625

[33] Ueda-Nakamura T., Mendonça-Filho R.R., Morgado-Díaz J.A., Maza P.K., Dias Filho B.P., Cortez D.A.G. (2006) Antileishmanial activity of Eugenol-rich essential oil from *Ocimum gratissimum*. Parasitol. Int. 55, 99–105 10.1016/j.parint.2005.10.006 16343984

[34] Borji H., Moghaddas E., Razmi G.H., Bami M.H., Mohri M. and Azad M. (2011) Prevalence of pigeon haemosporidians and effect of infection on biochemical factors in Iran. J. Parasit. Dis. 35, 199–201 10.1007/s12639-011-0056-1 23024504 PMC3235371

[35] Bennett G.F., Caines J.R. and Bishop M.A. (1988) Influence of blood parasites on the body mass of passeriform birds. J. Wildl. Dis. 24, 339–343 10.7589/0090-3558-24.2.339 3373640

[36] Bennett G., Peirce M. and Ashford R. (1993) Avian haematozoa: mortality and pathogenicity. J. Nat. Hist. 27, 993–1001 10.1080/00222939300770621

[37] Ots I. and Horak P. (1988) Health impact of blood parasites in breeding great tits. Oecologia 116, 441–448 10.1007/s004420050608 28307512

[38] Manwell R.D. and Loeffler C.A. (1961) Glucose consumption by *Haemoproteus columbae*. J. Parasitol. 47, 285–290 10.2307/3275308 13766354

[39] Mushi E.Z., Binta M.G., Chabo R.G., Mathaio M. and Ndebele R.T. (1999) *Haemoproteus columbae* in domestic pigeons in Sebele, Gaborone, Botswana. Onderstepoort J. Vet. Res. 66, 29–32 10396759

[40] Archawaranon M. and Subinprasert S. (2005) Bird-parasite relations: a hill mynah case study. J. Entomol. 2, 112–116 10.3923/je.2005.112.116

[41] Mubarak M. and Abed G.H. (2005) Pathological changes of lung tissues of pigeons (*Columba livia domestica*) infected with *Haemoproteus columbae* (Haemosporina: Haemoproteidae). J. Biol. Sci. 5, 536–541 10.3923/jbs.2005.536.541

