# Supplementary material for: Effects of Eugenol on Haemoproteus columbae in domestic pigeons (Columba livia domestica) from Riyadh, Saudi Arabia
Source: Biosci Rep. 2019 May 24;39(5):BSR20190409. doi: 10.1042/BSR20190409 (PMC6533204; doi:10.1042/BSR20190409)
Supplement: Supplementary file 1 [file bsr20190409_Supp1.pdf]

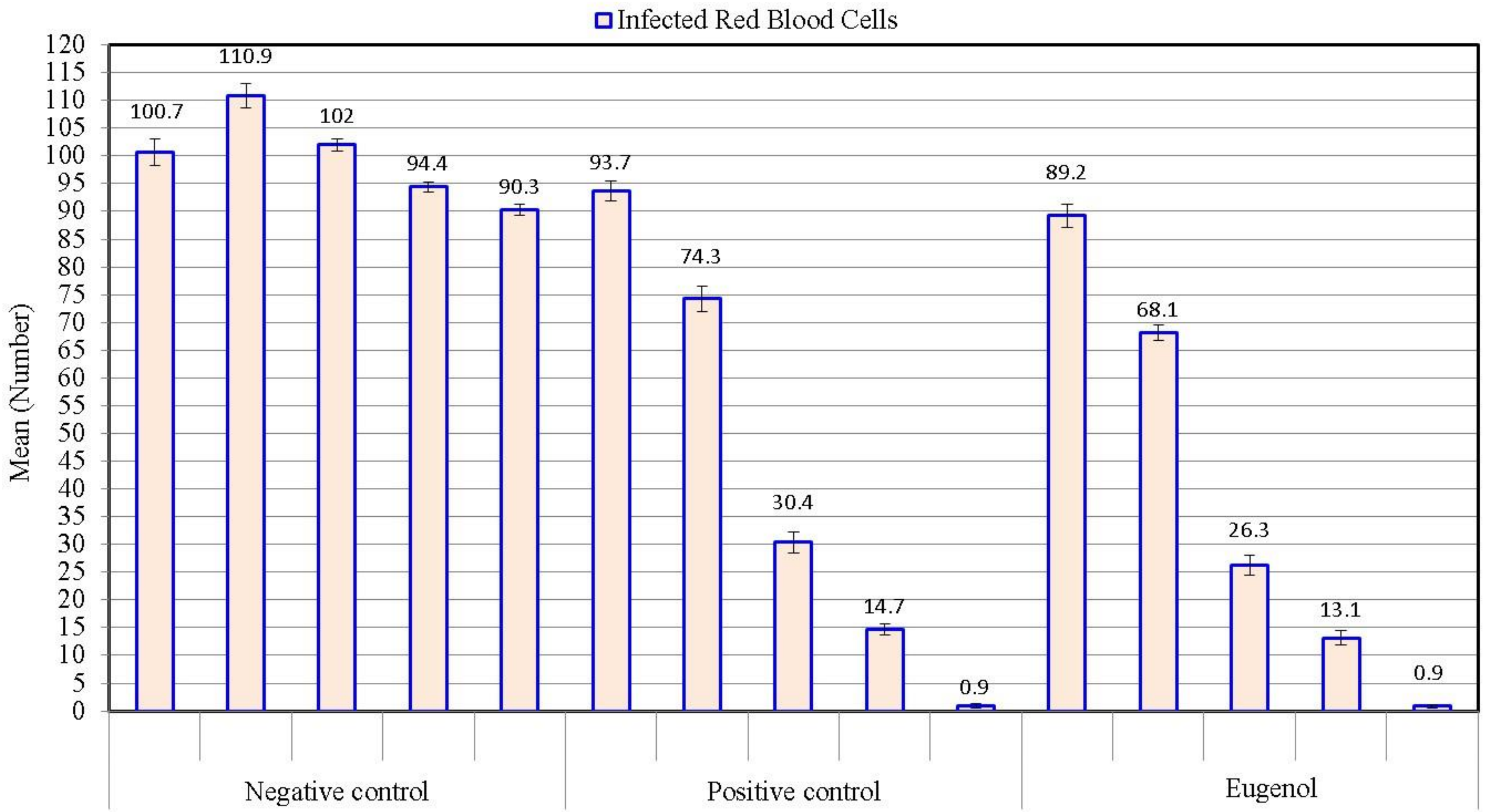

Mean values interaction between treatment and times on the infected red blood cells number in of squabs.
